# The Association Between Spousal Caregiver Status and Psychological Well-Being Among Older Adults

**DOI:** 10.1093/geroni/igaa057.095

**Published:** 2020-12-16

**Authors:** Yang Wang

**Affiliations:** University of Illinois at Urbana-Champaign, Urbana, Illinois, United States

## Abstract

Older adults often bear the responsibility of taking care of their spouses who have physical or cognitive impairments. Although previous studies suggested various caregiving-related negative experiences might be related to older spousal caregivers’ mental health status, the components of psychological well-being (PSW) among older spousal caregivers have not been fully explored. This study examined the association between spousal caregiver status and PSW. Data were drawn from wave 2014 of the Health and Retirement Study. The sample consisted of 3,857 adults who were above 50, and 376 of the participants provided care in activities in daily life (ADL) or instrumental activities in daily life (IADL) to their spouses/partners. Three hundred and thirty-one of them had a spouse/partner who needed care, but did not provide the care to their spouse/partner. The majority of participants, 3,150 of them, did not provide care, and their spouse/partner did not need care. Four domains of PSW were assessed: purpose of life, life satisfaction, positive affect, and negative affect. Univariate analyses and multivariable linear regression were used for analyses. Spousal caregivers and non-caregivers with need had a worse PS status, compared to non-caregivers without need. But after adjusting for socio-economic resources, health status, or social support factor, the difference in their four domains of PSW was insignificant. Older spousal caregivers’ PSW could be protected by promoting that population’s socio-economic resources and health status. Future practice needs to address caregivers’ emotional need. Future research need to examine the long-term effect of caregiving on older spousal caregivers’ PSW.

